# Supplementary material for: Use of Technology-Based Interventions in the Treatment of Patients with Overweight and Obesity: A Systematic Review
Source: Nutrients. 2020 Nov 26;12(12):3634. doi: 10.3390/nu12123634 (PMC7760174; doi:10.3390/nu12123634)
Supplement: Supplementary file 1 [file nutrients-12-03634-s001.pdf]

**Supplementary S1.** Weight lost according to the technology used.

| | | Nr of Groups that used it | Average weight loss ( kg) |
|---|---|---|---|
| **Smartphone** | App | 23 | 3.82 |
| | SMS | 11 | 3.07 |
| Virtual reality | | 4 | 4.7 |
| Website | | 4 | 3.75 |
| PDA or EJ | | 6 | 2.00 |
| PA monitor | | 4 | 6.21 |
| Online software | | 3 | 2.95 |
| DVD | | 1 | 0.48 |
